# Analysis of the oxidized low density lipoprotein receptor 1 gene as a potential marker for carcass quality traits in Qinchuan cattle

**DOI:** 10.5713/ajas.18.0079

**Published:** 2018-07-26

**Authors:** Lin-sheng Gui, Sayed Haidar Abbas Raza, Jianlei Jia

**Affiliations:** 1 State Key Laboratory of Plateau Ecology and Agriculture, Qinghai University, Xining, Qinghai 810016, China; 2 College of Agriculture and Animal Husbandry, Qinghai University, Xining, Qinghai 810016, China; 3 College of Animal Science and Technology, Northwest A&F University, Yangling, Shaanxi 712100, China

**Keywords:** Carcass Quality Traits, Qinchuan Cattle, Association Analysis, Oxidized Low-density Lipoprotein Receptor 1 Gene

## Abstract

**Objective:**

The oxidized low density lipoprotein receptor 1 (*OLR1*) gene plays an important role in the degradation of oxidized low-density lipoprotein and adipocyte proliferation in mammals. For this reason, we aimed at investigating the association of *OLR1* gene polymorphisms with carcass quality traits in Chinese Qinchuan cattle.

**Methods:**

The single nucleotide polymorphism (SNP) was identified in the 3′ untranslated region of bovine *OLR1* gene by DNA sequencing. In addition, the haplotype frequency and linkage disequilibrium estimates of three SNPs were evaluated in 520 individuals.

**Results:**

Results indicated that the studied three SNPs were within the range of moderate genetic diversity (0.25< polymorphism information content<0.5). Haplotype analysis of three SNPs showed that ten different haplotypes were identified, but only five haplotypes were listed as those with a frequency of <0.05 were excluded. The Hap3 (-G_1_T_2_C_3_-) had the highest haplotype frequency (42.10%). Linkage disequilibrium analysis showed that the three SNPs had a low linkage (*r*^2^<0.001). The T10588C and C10647T were significantly associated with backfat thickness and intramuscular fat content in Qinchuan cattle.

**Conclusion:**

Based on our results, we believe that the *OLR1* gene could be a strong candidate gene for influencing carcass quality traits in Qinchuan cattle.

## INTRODUCTION

In China, Qinchuan cattle (*Bos taurus*) are generally considered as efficient converters of poor quality roughage to meat [1]. However, the carcass quality traits, i.e., back fat thickness, loin muscle area (LMA) and intramuscular fat (IMF), are noticeably lower than those of other exotic commercial cattle breeds (such as Wagyu cattle) [2]. In the process of livestock breeding, carcass traits are a good tool to assess the economic value of animals, which, at least in part, are affected by genetic variability [3]. Therefore, to identify polymorphisms of effective genes associated with carcass quality traits would be significant for marker assisted selection programs for breed improvement.

The oxidized low-density lipoprotein receptor 1 (*OLR1*), also known as *LOX1*, is a type-II membrane cell-surface protein [4], that plays an important role in the degradation of oxidized low-density lipoprotein (*Ox-LDL*) and adipocyte proliferation [5]. The *OLR1* gene was first identified by Sawamura et al [6] and found to influence lipid metabolism in the liver and mammary glands [6,7]. In mouse adipose tissue, the over-expression of the *OLR1* gene may improve cholesterol content, free fatty acid intake and lipid drops [8]. In pig adipose tissue, the expression of *OLR1* was correlated with peroxisome proliferator-activated receptor gamma, fas cell surface death receptor, and sterol regulatory element binding transcription factor-1c, which demonstrated that *OLR1* gene was highly associated with fat deposition and its transcription [9]. As a core surface receptor, *OLR1* was reported to support bind, internalizes and proteolytic degrades *Ox-LDL*, thereby controlling glucose and lipid metabolism in the mammary gland [10,11].

Based on the role of *OLR1* gene in lipid metabolism, the objectives of this study were to investigate the genetic variations within *OLR1* gene in Chinese native breed, and to analyze the associations between genetic variations and carcass quality traits. Results gleaned from this study could possibly contribute to better breeding plan and policies.

## MATERIALS AND METHODS

All animal procedures were performed according to guidelines laid down by the China Council on Animal Care, and the protocols were approved by the Experimental Animal Manage Committee (EAMC) of Qinghai University.

A total number of 520 cows (aged 18 to 24 months) were randomly collected from the experiment farm of national beef cattle improvement center (Yangling, Shaanxi, China). All cows were kept under similar dietary and environmental conditions. Genomic DNA was extracted from whole blood samples using a Blood DNA Kit (OMGAM Bio-Tek, Doraville, USA). Meanwhile, ultrasound measurements were available for each individual, including backfat thickness, LMA and IMF content.

Available sequence information from bovine *OLR1* gene (Genbank accession no AC_000162) was used to design polymerase chain reaction (PCR) primers. Details of primer nucleotide sequences for all primers, PCR product lengths, primers locations and annealing temperatures are shown in Table 1. PCR was performed in a 20 μL reaction mixture containing 50 ng of genomic DNA, 10 pM of each upstream and downstream primer, 0.20 mM of dNTPs, 2.5 mM MgCl_2_ and 0.5 U Taq polymerase (TaKaRa, Dalian, China). The reaction conditions were as follows: initial denaturation of DNA for 5 min at 95°C, followed by 35 cycles at 94°C for 30 s, the annealing temperature 30 s, and 40 s at 72°C followed by a final extension of primer for 10 min at 72°C.

Gene frequencies, Hardy-Weinberg equilibrium (HWE), and polymorphism information content (PIC) were tested by POPGENE software package (Version 3.2). Linkage disequilibrium (LD) and haplotype construction was performed with the online version of SHEsis software [12]. Combinations with frequencies below 5.0% were not included, and the remaining combinations were selected for further analysis.

According to the pedigree information, the 520 daughters came from 6 different sires. In livestock species, this affinity may influence the genotype of offspring. So, the sire is the random effect in the model. Age factors were divided into four levels (18, 20, 22, and 24-month age periods, respectively) in the model, due to the longer fattening periods of Qinchuan cattle. The association of each single nucleotide polymorphism (SNP) marker genotype and carcass traits was studied using a general linear model (GLM) procedure implemented in SPSS 16.0 (IBM Company, NY, USA) software package. The mixed model equation used in the study was as follows:

$$Y_{ijk}=\mu +G_{i}+A_{j}+S_{k}+e_{ijk}$$

Where *Y_ijk_* is the trait measurement, *μ* is the overall mean, *G_i_* is the fixed effect of genotype (i = 1, 2, and 3), *A_j_* is the fixed covariate of age (j = 1, 2, 3, and 4), *S_k_* is the random effect of sire (k = 1 through 6) and *e_ijk_* is the random error.

For a more detailed review of the results, we also corrected p values via the Bonferroni correction [13]. This correction was used to account for multiple tests and yielded more robust results. Differences were considered significant if p<0.05. Data are expressed as the mean±standard error.

## RESULTS

### Investigation of polymorphic loci on the bovine *OLR1* gene

**Figure 1:**
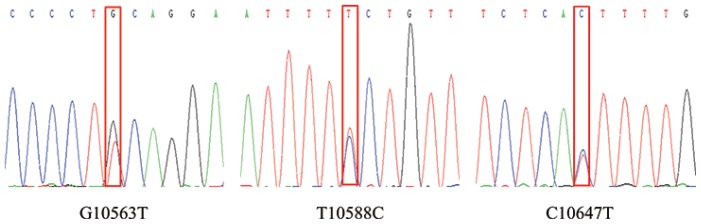
The sequencing peak of three single nucleotide polymorphisms (SNPs) from oxidized low density lipoprotein receptor 1 (*OLR1*) gene.

In this study, three SNPs (G10563T, T10588C, and C10647T) were identified by pool DNA sequencing in bovine *OLR1* gene (Figure 1). Table 2 illustrates the genotypic and allelic frequencies at all loci of bovine *OLR1* gene. The G10563T-A allele, T10588C-A allele, and C10647T-C allele were found to be predominant in the studied samples. In this study, the calculated PIC values for each locus ranged from 0.258 to 0.328. According to the convention for classification of PIC, three SNPs were within the range of moderate genetic diversity (0.25<p<0.50). Besides, the chi-square test showed that those SNPs were in HWE.

### Linkage disequilibrium within bovine *OLR1* gene

As shown in Table 3, the Hap3 (-G_1_T_2_C_3_-, 42.10%) occurred with greater frequency than the others in Qinchuan cattle. The values of *D*′ varied from 0.019 to 0.094, and the *r*^2^ values were from 0.000 to 0.001, indicating that those SNPs had low LD. It could be argued that recombination will be high, and LD will be low in genovariation-dense regions.

### Effects of different genotypes of *OLR1* gene SNP loci on carcass quality traits

The association results are presented in Table 4. At the T10588C locus, the influence of TT genotype resulted in the highest mean for backfat thickness compared to animals with genotype CC in Qinchuan cattle (p<0.05). At the C10647T locus, individuals carrying the TT genotype had significantly greater backfat thickness than those with CC genotype (p<0.05). At the G10563T locus, genotypes did not show any significant correlation with carcass quality traits (p>0.05).

Table 5 shows the association of selected haplotypes (with frequencies of 5% or more) with carcass quality traits. When the combination results were compared no significant differences were detected between the combined haplotype of these SNPs and carcass quality traits in the Qinchuan cattle population (p>0.05).

## DISCUSSION

The *OLR1* gene was reported to possess complex biological functions [14], especially regarding degradation of low-density lipoprotein and glucose metabolism in liver. Previous studies have shown that genetic polymorphisms in the *OLR1* gene are associated with economic traits in livestock. Khatib et al [3] reported that the SNP A8232C in the 3′ untranslated region (UTR) of *OLR1* gene was associated with *OLR1* gene expression, and linked to significant effects on milk fat yield and fat percentage in Holstein dairy cattle. Similarly, one novel SNP (C223A) detected in *OLR1* gene was associated with milk fat percentage in Polish Holstein-Friesian bulls [15]. Michael et al [16] showed that one novel SNP (c.-495T>C) in promoter region of *OLR1* gene had a strong effect on rib-eye area in Angus steer population. The research of Fonseca et al [17] showed that a marker (rs109019599) on *OLR1* gene was associated with rump fat thickness and weaning weight in Nelore cattle. Based on these outcomes, it is our belief that the *OLR1* gene could be an excellent candidate gene for fat deposition-related traits in livestock.

The bovine *OLR1* gene localizes on chromosome 18, has 6 exons [11], and is highly expressed in lung, liver and adipose tissue [9]. In the current study, we detected three SNPs in 3′ UTR, including G10563T, T10588C, and C10647T. Data analysis revealed that the individuals with genotype TT of T10588C and C10647T had significantly greater backfat thickness and IMF content than those with genotype CC, demonstrating that allele T might be associated with an increase in backfat thickness and IMF content in Qinchuan cattle. Therefore, we suggest that the selective breeding of the heterozygotes at both T10588C and C10647T loci of *OLR1* in Qinchuan cattle may improve on the carcass quality traits.

The 3′ UTR miRNA-related SNPs may be within or at the vicinity of the miRNA binding site, which could impair regulatory functions of the associated miRNA [18,19]. This could result in variations in the level or timing of gene expression, ultimately affecting phenotypes [20,21], as has been previously demonstrated in Duroc pigs [22], Polish Holstein-Friesian [23], and Guanzhong dairy goats [24]. In the current paper, bioinformatics analysis was used to predict the effects of the two SNPs in the 3′ UTR of the *OLR1* gene on the miRNA binding sites using the online version of Targetscan software (http://www.targetscan.org/vert). The data indicated that allele C in T10588C, and allele T in C10647T altered the base within binding sites of bta-miR-12047 and bta-miR-12021, respectively. Thus, it can be reasonably inferred that the mutations may alter *OLR1* gene expression level by modifying miRNA binding sites within the 3′ UTR. As a result, the mutations could affect fatty acid metabolism in Qinchuan cattle.

In summary, three polymorphisms in the *OLR1* were identified in Qinchuan cattle. The association analysis of single markers (T10588C and C10647T) revealed prominent effects on carcass quality traits. However, further research and validation of various allelic effects, functional mechanisms and bioactivity are needed in larger population to explore the usage of *OLR1* gene in beef cattle breeding.

## Figures and Tables

**Table 1:** Primer sequences used in PCR assays with *OLR1* gene fragments

Primer name	Primer sequence (5′ to 3′)	Annealing temperature (°C)	Product length (bp)	Amplified region
L1	ACTCCAGCAGGAACTCACA AATGATAAGCCAACTTGGT	56.7	336	Exon 1 and intron 1
L2	ATATCTGATATTGAATCCCA ATGAGGGCTTATAAACA	60.0	381	Intron 1, exon 2, and intron 2
L3	TTATTGGGAATTGGAATTGG CTCTATTTTTGTCATCCT	57.2	448	Intron 2, exon 3, and intron 2
L4	GTTAAGAATTGTAGAAATAT GACGCCCCACTTGTAAG	63.5	653	Intron 3, exon 4, intron 4, and exon 5
L5	AAGGCGAATCTATTGAGAGC CCTAGAAGAAAGCATAGGAC	60.4	360	Exon 6 and 3′UTR

PCR, polymerase chain reaction; *OLR1*, oxidized low density lipoprotein receptor 1, UTR, untranslated region.

**Table 2:** Distribution of genotype and allele frequencies in Qinchuan cattle

Site	Genotype (N)	Genotypic frequency (%)	Alleles	Allele frequency (%)	χ^2^ (HWE)	PIC
G10563T	GG (348)	66.92	G	81.25	p>0.05	0.2583
rs722568839	GT (149)	28.65	T	18.75		
	TT (23)	4.43	-	-		
T10588C	TT (290)	55.77	T	74.04	p>0.05	0.3105
rs132917098	TC (190)	36.54	C	25.96		
	CC (40)	7.69	-	-		
C10647T	CC (268)	72.12	C	70.77	p>0.05	0.3281
rs45938133	CT (200)	24.23	T	29.23		
	TT (52)	3.65	-	-		

χ^2^ (HWE), Hardy-Weinberg equilibrium χ^2^ value, Hard-Weinberg equilibrium (p>0.05), Hardy–Weinberg disequilibrium (p<0.05).

**Table 3:** Haplotypes of *OLR1* gene and their frequencies in Qinchuan cattle1)

Haplotype	G10563T	T10588C	C10647T	Frequency (%)
Hap1	G1	C2	C3	15.70
Hap2	G1	C2	T3	5.90
Hap3	G1	T2	C3	42.10
Hap4	G1	T2	T3	17.60
Hap5	T1	T2	C3	9.80

*OLR1*, oxidized low density lipoprotein receptor 1.

1) C_1_ and T_1_ were haplotypes of G10563T, C_2_ and T_2_ were haplotypes of T10588C, C_3_ and T_3_ were haplotypes of C10647T.

**Table 4:** Association of carcass traits with marker genotypes within OLR1 gene in Qinchuan cattle

Site	Trait	Genotype (mean±standard error)			p value1)
		GG	GT	TT	
G10563T	BT (cm)	0.981±0.017	0.963±0.026	1.031±0.066	0.902
rs722568839	LMA (cm^2^)	64.195±0.467	64.367±0.714	61.753±1.817	0.543
	IMF (%)	7.138±0.041	7.207±0.063	7.482±0.161	0.338
		TT	TC	CC	
T10588C	BT (cm)	0.987±0.019^a^	0.991±0.023^a^	0.850±0.050^b^	0.031^d^
rs132917098	LMA (cm^2^)	63.541±0.511	64.757±0.631	65.504±1.376	0.404
	IMF (%)	7.264±0.045	7.146±0.055	6.639±0.120	0.177
		CC	CT	TT	
C10647T	BT (cm)	0.907±0.019^b^	1.005±0.021	1.240±0.042^a^	0.013^d^
rs45938133	LMA (cm^2^)	64.720±0.532	63.608±0.616	63.159±1.207	0.517
	IMF (%)	7.084±0.047	7.223±0.054	7.440±0.106	0.069

*OLR1*, oxidized low density lipoprotein receptor 1; BT, backfat thickness; LMA, loin muscle area; IMF, intramuscular fat content.

1) Probability of the F-test for genotype effect.

a,b Means with different superscripts are significantly different (p<0.05).

d Significant effect (p<0.05) after modified Bonferroni correction for trait-wise multiple tests.

**Table 5:** Associations of carcass traits with diplotypes within *OLR1* gene in Qinchuan cattle

Haplotypes	Hap3/1 (67)	Hap3/2 (41)	Hap3/3 (94)	Hap3/4 (81)	Hap3/5 (48)	p-value1)
BT (cm)	0.921±0.037	1.055±0.047	0.907±0.031	1.027±0.034	0.984±0.044	0.085
LMA (cm^2^)	64.347±1.084	63.905±1.385	64.476±0.915	63.347±0.986	64.904±1.280	0.773
IMF (%)	7.021±0.094	7.070±0.120	7.186±0.079	7.293±0.085	7.127±0.110	0.254

*OLR1*, oxidized low density lipoprotein receptor 1; BT, backfat thickness; LMA, loin muscle area; IMF, intramuscular fat content.

1) Probability of the F-test for genotype effect.
